# Characterization of the Humoral Immune Response Induced after Infection with Atypical Porcine Pestivirus (APPV)

**DOI:** 10.3390/v11100880

**Published:** 2019-09-21

**Authors:** Gökce Nur Cagatay, Denise Meyer, Michael Wendt, Paul Becher, Alexander Postel

**Affiliations:** 1 Department of Infectious Diseases, Institute of Virology, University of Veterinary Medicine, 30559 Hannover, Germany; 2 Clinic for Swine, Small Ruminants and Forensic Medicine, University of Veterinary Medicine, 30173 Hannover, Germany

**Keywords:** pestivirus, APPV, humoral immune response, ELISA, virus neutralization test

## Abstract

Atypical porcine pestivirus (APPV) is a widely distributed pathogen causing congenital tremor (CT) in piglets. So far, no data are available regarding the humoral immune response against APPV. In this study, piglets and their sows from an affected herd were tested longitudinally for viral genome and antibodies. APPV genome was detected in the majority of the piglets (14/15) from CT affected litters. Transient infection of gilts was observed. Kinetics of E^rns^- and E2-specific antibodies and their neutralizing capacity were determined by recently (E^rns^) and newly (E2) developed antibody ELISAs and virus neutralization assays. Putative maternally derived antibodies (MDA) were detected in most piglets, but displayed only low to moderate neutralizing capacity (ND_50_ ≤ 112). Horizontal APPV transmission occurred when uninfected and infected piglets were mingled on the flat deck. Horizontally infected piglets were clinically inapparent and showed only transient viremia with subsequently consistently high E2 antibody levels. For piglets from CT affected litters, significantly lower neutralizing antibody titers were observed. Results indicate that E2 represents the main target of neutralizing antibodies. Characterization of the humoral immune response against APPV will help to provide valuable serological diagnosis, to understand the epidemiology of this novel pathogen, and to implement tailored prevention strategies.

## 1. Introduction

Pestiviruses belong to the family *Flaviviridae* and comprise animal pathogens associated with severe economic losses in livestock breeding [1]. The genus *Pestivirus* consists of at least eleven species (*Pestivirus A* to *K*), of which four are well established pestiviruses (*Pestivirus A* to *D*), namely bovine viral diarrhea virus 1 (BVDV-1), bovine viral diarrhea virus 2 (BVDV-2), classical swine fever virus (CSFV) and border disease virus (BDV) [2]. Atypical porcine pestivirus (APPV) is a genetically distinct pestivirus, which was discovered in 2015 and is classified as *Pestivirus K* species [2,3]. APPV was demonstrated to be one major causative agent of congenital tremor (CT) [4,5,6,7,8,9,10,11]. When present, discrete histopathological alterations can be found mainly in the central nervous system. APPV-induced CT manifests as tremors of the head and limbs of new born piglets [6,9,12]. APPV is likely to be present worldwide and highly prevalent in domestic pig populations [3,13,14,15,16,17,18,19,20,21,22,23]. Recently, wild boar were identified to serve as a wild animal reservoir for APPV, which might be of epidemiological relevance [24,25]. Economic losses caused by APPV infection can occur due to elevated pre-weaning mortality [9].

Pestiviruses possess three envelope glycoproteins, namely E^rns^, E1 and E2, which are involved in virus attachment and entry into target cells [1]. Pestivirus glycoproteins E^rns^, E2 and the non-structural protein 3 (NS3) are known to provoke a detectable humoral immune response in a host after a natural infection [26]. In classical pestiviruses, such as CSFV and BVDV, E2 functions as the immune-dominant antigen and is the main target for neutralizing antibodies that confer protective immunity to the host [27,28]. E^rns^ is also known to induce neutralizing antibodies, but to a much lower extent [29]. However, so far nothing is known regarding the induction of protective immunity by structural proteins of APPV. In addition to low sequence homology, the predicted E2 protein of APPV is significantly shorter compared to classical pestiviruses, thus likely to show major differences in structure [3]. Recombinant APPV E2 glycoprotein was recently shown to induce humoral immune response in mice [30]. Yet, there are no studies available characterizing the humoral immune response during natural APPV infection courses in the porcine host.

In this study, dynamics of APPV field infection were monitored for several months (from birth to slaughter) and kinetics of induced E^rns^ and E2-specific antibodies, including their neutralizing capacity, were determined. The aim of this study is to provide insight into the humoral immune response against APPV infections including the neutralizing capacity of induced antibodies. Findings will contribute to the understanding of the epidemiology of this novel pathogen and will help to implement tailored prevention strategies.

## 2. Materials and Methods

### 2.1. Origin of Samples

**Figure 1:**
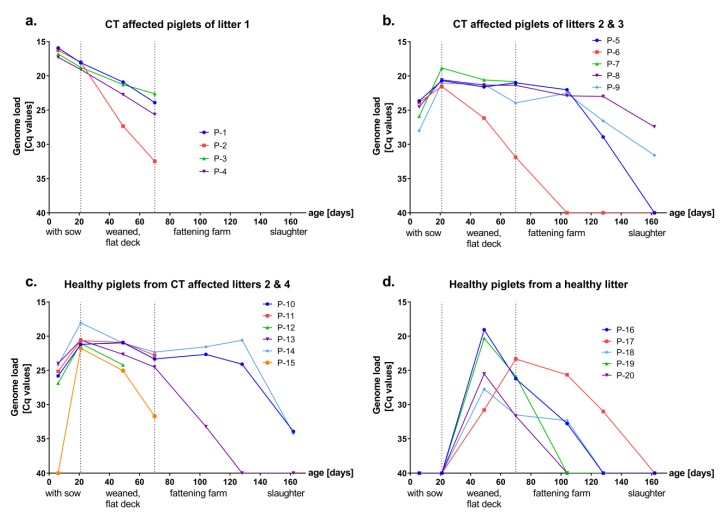
Atypical porcine pestivirus (APPV) genome titers of piglets from congenital tremor (CT) affected litters (**a–c**) and piglets from a healthy litter (**d**). Genome loads of 20 piglets were investigated by real-time RT-PCR. Cq values of serum samples (*y* axis), the age of piglets and holding conditions (*x* axis) are indicated. Vertical dashed lines indicate the changes in the holding conditions. Piglets from CT affected litter 1 (**a**) were cross-fostered immediately after birth to a pluriparous sow with a healthy litter. Piglets from CT affected litters 2, 3 and 4 are grouped into two panels (**b,c**) by the occurrence of CT.

**Figure 2:**
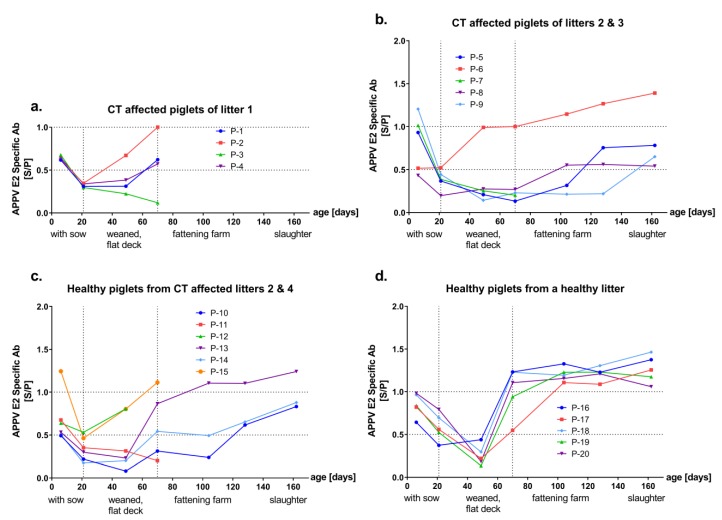
Kinetics of E2-specific antibodies induced after APPV infection. APPV E2-specific antibody levels of 20 pigs were investigated by E2 ELISA and results are given as S/P values (S/P ≤ 0.5, low; S/P = 0.5–1.0 intermediate; S/P ≥ 1.0 high reactivity). Horizontal dashed lines indicate the APPV E2-specific Ab levels as low, intermediate and high. Vertical dashed lines indicate the changes in the holding conditions. Piglets from CT affected litter 1 (**a**) were cross-fostered immediately after birth to a pluriparous sow with a healthy litter. (**d**) Piglets from CT affected litters 2, 3 and 4 are grouped into two panels (**b,c**) by the occurrence of CT.

**Figure 3:**
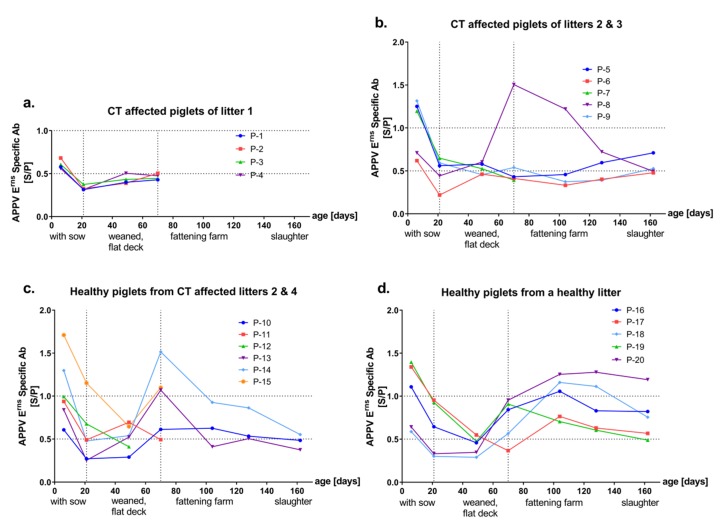
Kinetics of E^rns^-specific antibodies induced after APPV infection. APPV E^rns^-specific antibody levels of 20 pigs were investigated by E^rns^ ELISA and results are given as S/P values (S/P ≤ 0.5, low; S/P = 0.5–1.0 intermediate; S/P ≥ 1.0 high reactivity). Horizontal dashed lines indicate the APPV E^rns^-specific Ab levels as low, intermediate and high. Vertical dashed lines indicate the changes in the holding conditions. Piglets from CT affected litter 1 (**a**) were cross-fostered immediately after birth to a pluriparous sow with a healthy litter. (**d**) Piglets from CT affected litters 2, 3 and 4 are grouped into two panels (**b,c**) by the occurrence of CT.

For detection of APPV genomes and APPV-specific antibodies, serum samples of 20 piglets and six first parity sows with CT affected litters were taken serially at a breeding farm located in North Rhine-Westphalia, Germany, during a CT outbreak in 2016. Only the litters from first parity sows were affected with CT on this farm (*n* = 15, litter 1 to 4, Figure 1, Figure 2 and Figure 3, panels a–c). A majority of the piglets originated from CT affected litters (*n* = 9/15, litters 1, 2 and 3, Figure 1, Figure 2 and Figure 3, panels a and b) showed clinical signs of CT, whereas some littermates were apparently healthy (*n* = 6/15, litters 2 and 4, Figure 1, Figure 2 and Figure 3, panel c). Piglets from CT affected litter 1 (P-1 to P-4, Figure 1, Figure 2 and Figure 3, panel a) were cross-fostered to a pluriparous sow immediately after birth. One healthy litter of a pluriparous sow (*n* = 5, Figure 1, Figure 2 and Figure 3, panel d) was also sampled.

All piglets and gilts were sampled at tree time points starting on the sixth day post natum (day 6, 21 and 48, Figure 1, Figure 2 and Figure 3). After being weaned at an age of 21 days, all 20 piglets (infected and uninfected) were mingled on the flat deck until they were sent to a fattening farm on day 69 (Figure 1, Figure 2 and Figure 3). Most of the piglets (*n* = 12) were sampled three additional times until slaughter at 161 days of age (day 103, 127, 161, Figure 1, Figure 2 and Figure 3). At all samplings, health status of the pigs was monitored by a veterinarian. All samples were taken according to the national legislation and the animal welfare act. Initial samples were taken for diagnostic purposes. Follow-up sampling was ethically approved by the local authorities of North Rhine-Westphalia (Landesamt für Natur, Umwelt und Verbraucherschutz Nordrhein-Westfalen; LANUV) under the permission number 84–02.05.40.16.020.

### 2.2. RNA Purification and Real-Time RT-PCR

RNA extraction was performed using the QIAamp Viral RNA Mini Kit (Qiagen, Hilden, Germany) according to manufacturer’s protocol. For the detection of APPV, a previously described pair of NS3-specific primers (APPV_CT-59_5587-fw, APPV_CT-59_5703-rev) was used, which has been demonstrated to be broadly reactive within distinct APPV [6]. One-step SYBR Green real-time RT-PCR was performed using the Mx3005P Q PCR System (Agilent Technologies, Santa Clara, USA) and QuantiTect^®^ SYBR^®^ Green RT-PCR Kit (Qiagen, Hilden, Germany) according to the manufacturer’s recommendations. The assay was performed using the following thermal program: 50 °C for 10 min (RT-step), 95 °C for 15 min and 40 cycles of 95 °C for 15 s, 56 °C for 30 s, 72 °C for 30 s and 79 °C for 15 s. Melting curve analysis was used for evaluation of the results and specificity of the amplification was confirmed by gel electrophoresis.

### 2.3. Conventional RT-PCR and Nucleotide Sequencing

To determine the genotype of the APPV causative for the investigated outbreak, a highly genome-positive sample from a six-day-old piglet (Cq value 15) was subjected to nucleotide sequencing. Amplification of an 806 bp partial NS3 coding sequence was performed as previously described, using the primers APPV_5030-fw, APPV_5835-rev [6]. Sanger sequencing was conducted by LGC genomics (Berlin, Germany). A sequence was generated using the GENtle software (version 1.9.4.0) and the obtained consensus sequence was subjected to BLAST search (NCBI; National Center for Biotechnology Information).

### 2.4. Indirect APPV-Specific ELISA Assays

For antibody detection in serum samples, an indirect APPV-specific enzyme-linked immunosorbent assay (ELISA) based on glycoprotein E^rns^ was performed as described previously [31].

With a similar approach, an additional indirect APPV-specific ELISA based on glycoprotein E2 was developed. Glycoprotein E2 of the German APPV isolate Ger-NRW_L277 (GenBank MF167291.1) was expressed in *Leishmania tarentolae* using the LEXSYcon2 Expression Kit (Jena Bioscience, Jena, Germany). The C-terminal transmembrane domain of the glycoprotein E2 was trimmed and the coding sequence region from position 2101 to position 2733 (GenBank MF167291.1) was cloned into the pLEXSY-hyg2 plasmid in frame with the N-terminal LMSAP1 signal peptide for secretory expression and a C-terminal His-tag for affinity purification. Secreted antigen was collected and purified in ÄKTA™ Pure Chromatography System using HisTrap™ Excel purification column (GE Healthcare, Uppsala, Sweden). Purified E2 protein was tested in immunoblot and one specific protein band was detected using both, an APPV antibody positive sera (diluted 1:50) and a His-tag specific antibody (Roche diagnostics, Mannheim, Germany, diluted 1:100). Antigen was diluted in coating buffer (0.1 M NaCO_3_ + 0.1 M NaHCO_3_, pH 9.6) and 0.3 µg per well coated onto Nunc™ MediSorp™ 96-well ELISA plates (Thermofisher Scientific, Roskilde, Denmark) at 4 °C, overnight. Blocking was performed with PBS containing 0.05% Tween 20 and 4% skim milk powder at room temperature for 2 h.

Both E2 and E^rns^ ELISA assays were performed by incubating 1:25 diluted samples at 37 °C for 1 h. Specific binding of the antibodies was detected by anti-pig IgG (whole molecule)-peroxidase (A5670, Sigma-Aldrich, MO, USA, diluted 1:35,000) and 3,3’,5,5´Tetramethylbenzidine (TMB, Sigma-Aldrich, MO, USA) according to the manufacturer’s protocol.

Serological statuses of the samples are given as S/P (sample/positive control) values for both ELISA assays, in order to achieve better inter-assay comparability. Subsequently, APPV antibody status was classified into low (S/P ≤ 0.5), intermediate (0.5 < S/P < 1.0), or high reactivity (S/P ≥ 1.0) as described previously [31]. All tested sera were investigated in duplicates and mean values were used for S/P calculations. Duplicates showing a variation higher than 10% were repeated.

### 2.5. Cells and APPV Stock Used for Virus Neutralization Tests

The porcine kidney cell line SPEV (cell line 0008, Collection of Cell Lines in Veterinary Medicine, FLI, Germany) was maintained as a monolayer in Earle´s minimal essential medium (EMEM) containing 5% fetal bovine serum (FBS) and infected with APPV isolate Ger-NRW_L277 (GenBank MF167291.1). Continuous passaging of a cell culture persistently infected with APPV resulted in improved viral replication and increased titers. Determination of the complete polyprotein coding sequence by next generation sequencing revealed 10 nonsynonymous mutations in various regions of the viral genome on 100th passage (L277_p100_, Figure S2). The effect of these mutations, some of which are located in envelope glycoprotein regions, remains unclear and will be investigated in future studies. To obtain APPV stocks suitable for VNT, persistently infected cells were grown four days and supernatant was harvested together with the cells and stored at −80 °C in aliquots. For application in VNT, virus stocks were thawed and cell debris was removed by centrifugation (12,000× *g*, 10 min). The virus titer was determined by end point dilution assay and reached approximately 8 × 10^4^ TCID_50_/mL.

### 2.6. Neutralizing Capacity of APPV-Specific Antibodies

A virus neutralization test (VNT) was established according to a protocol that is routinely used in the EU and OIE Reference Laboratory for Classical Swine Fever for determination of antibody titers against classical pestiviruses [32]. Briefly, sera were complement inactivated for 30 min at 56 °C and tested in triplicates in a two-fold dilution series between 1:4 and 1:8,192. The APPV L277_p100_ test virus dilution was adjusted to 100 TCID_50_ (50% tissue culture infectious dose) per well (50 µL) and added to each well containing the diluted serum samples for an incubation (37 °C for 1 h). After the incubation, 1 × 10^4^ SPEV cells were added to each well and incubated at 37 °C for additional 72h. Since APPV does not cause any cytopathogenic effect in cell culture, immunofluorescence staining of the viral antigen was performed for visualization. For this purpose, a porcine field serum (diluted 1:2000) was used, which was demonstrated to be reactive against the recombinant APPV E2 and E^rns^ antigens in western blot and also served as a positive control in both ELISA assays. In each test, back titration of the virus was performed and one APPV antibody positive and one negative serum were included as controls (Figure S2). The test was regarded as valid if the negative control displayed no neutralizing effect and the APPV test virus was within the range of 30–300 TCID_50_ per well (50 µL). Spearman–Kaerber formula was used to calculate the neutralizing doses (ND_50_).

## 3. Results

### 3.1. Presence and Kinetics of APPV Genome in Healthy and CT Affected Piglets

Newborn piglets (*n* = 20) from individual litters and six gilts were investigated for APPV infection, presence of APPV-specific antibodies and their neutralizing capacity. All samples were tested for the presence of APPV genome by a real-time RT-PCR assay targeting the NS3 coding region. Two of six gilts were APPV genome positive at the first sampling on the sixth day post partum (Cq value 30 and 32), of which one remained positive at the following sampling (day 21, Cq value 34). All other samples from the gilts (until last sampling on day 48) were APPV genome negative.

With only one exception, piglets from CT affected litters (*n* = 15) were APPV genome positive from the first sampling at six days of age (Cq values between 15 and 27, Figure 1a–c). The remaining piglet (P-15) was found to be positive at the second sampling with an age of 21 days, which was the last day of piglets remaining with the sow (Figure 1c). Clinical signs of CT were observed in 9 of 15 piglets (P-1 to P-9, Figure 1a,b), which originated from affected litters. Three of the piglets affected with CT at birth (P-6, P-8, P-9, Figure S1) showed mild symptoms of tremor (head and ears) until slaughter, whereas the others remained without symptoms after weaning. Healthy piglets from a healthy litter (*n* = 5, P-16 to P-20, Figure 1d) were APPV genome negative from birth until weaning at 21 days of age. All initially uninfected piglets were APPV genome positive at 48 days of age, indicating horizontal infection with APPV during the first four weeks after mingling with the genome positive piglets on the flat deck (Cq values between 19–31, Figure 1d). No clinical signs were observed after horizontal infection with APPV. All sampled piglets (*n* = 20) were APPV genome positive on day 69, when they were sent to the fattening farm. Most of the piglets (*n* = 12/20) were sampled three additional times in the fattening farm, until slaughter at 161 days of age (day 103, 127, 161, Figure 1b–d). From the initially APPV infected pigs that were followed up until slaughter, the majority (*n* = 4/7) remained genome positive, suggesting persistent infections (P-8, P-9, P-10, P-14, Figure 1b,c). Viral genome loads declined only slowly over a period of several months. In contrast, all horizontally infected piglets (*n* = 5, P16 to P-20) were already genome negative at the time of slaughter (day 161) indicating a transient infection.

To determine the genotype of the APPV isolate causing the outbreak, a partial NS3 coding sequence (806 bp) from a highly genome positive sample (P-1, Cq value 15) was determined. Sequence analysis showed 100% nucleotide identity to the APPV isolate Ger-NRW_L277, which originated from the same region and year and was used as test virus in VNT.

### 3.2. Detection of Antibodies against the Envelope Proteins E^rns^ and E2 of APPV

All samples were investigated for the presence of APPV-specific antibodies in two ELISAs based on the E^rns^ or E2 envelope proteins of APPV, respectively. Sera of all gilts showed consistent levels of antibodies (intermediate to high) against both E2 and E^rns^ antigens. In general, various patterns of antibody response were observed in piglets from CT affected litters whereas a consistent antibody response was evident after a horizontal transient infection. Independent of the health status, all except one of the samples from six-day-old piglets (*n* = 19/20) showed intermediate to high reactivity against both E2 and E^rns^ antigen, which declined over time and reached minimal antibody titers at an age of 21 or 48 days (Figure 2 and Figure 3, panels a–d). At later time points, piglets from CT affected litters showed various levels of E2 and E^rns^-specific antibodies (Figure 2 and Figure 3, panels a–c). Consistent and high levels of antibodies against APPV E2 were observed from all horizontally infected piglets, at day 69 and later time points (P-16 to P-20, Figure 2d).

At the individual animal level (Figure S1), one piglet from a CT affected litter (P-15) revealed to have the highest E2 and E^rns^ antibody titers at an age of six days, which was APPV genome negative at this age and became infected before entering the flat deck (Figure 2c, Figure 3c and Figure S1). Few sera from individual animals were highly reactive only against the E2 or the E^rns^ antigen. Sera of two diseased piglets (P-2 and P-6) from different litters showed high reactivity against E2 (S/P value = 1) but not E^rns^ (S/P value = 0.5 and 0.4) at day 69 (Figure 2a,b and Figure S1). Only one of these piglets (P-6) was sampled also at later time points and the serum remained highly reactive against E2 until slaughter at day 161 (Figure 2b). Additionally, on day 69, another CT affected piglet (P-8) showed a transient high serum reactivity against E^rns^ (S/P value = 1.5), but not against E2 (S/P value = 0.2, Figure 3b and Figure S1).

### 3.3. Neutralizing Capacity of APPV-Specific Antibodies

**Figure 4:**
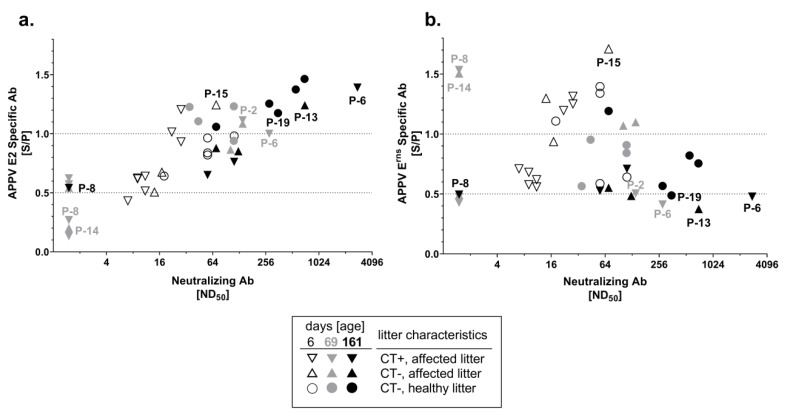
Reactivity of the sera against the APPV E2 and E^rns^ envelope proteins and virus neutralizing capacity. Symbols indicate the health and infection status of the piglets. Shades of the symbols indicate the age of piglets. (**a**) Correlation of APPV E2 antibody ELISA (*y* axis) and VNT results (*x* axis). (**b**) Correlation of APPV E^rns^ antibody ELISA (*y* axis) and VNT results (*x* axis). Individual animals indicated in the figures were described in detail within the results section. P-2, P-6, P-8, P-13, P-14 and P-15 are individually presented in Figure S1.

Neutralizing capacity of the porcine sera was analyzed with the newly established APPV VNT. Selected sera of piglets (*n* = 42) at an age of six, 69 and 161 days and from the gilts (*n* = 18) taken at six, 21 and 48 days after farrowing were analyzed for their neutralizing capacity. Gilts showed neutralizing antibody titers (ND_50_) between 28 and 112 and the individual titers remained consistent within approximately seven weeks (48 days) after farrowing. No major differences were observed between the gilts that were APPV genome positive and those being negative at the first sampling date. Analysis of serum samples from the six-day-old piglets (*n* = 16) resulted in detection of neutralizing antibody titers at similar level to the gilts, showing ND_50_ between nine and 112 (Figure 4). The serum of the only initially APPV genome negative piglet (P-15) from a CT affected litter showed a neutralizing antibody titer of 70 at day six, which is in line with high antibody levels determined in both ELISAs (Figure 4 and Figure S1). The majority of tested sera from 69-day-old pigs from CT affected litters (*n* = 6/10 tested sera) showed no neutralizing capacity (ND_50_ ≤ 4). Only two CT affected piglets (P-2 and P-6) from different litters developed neutralizing antibodies before transfer to the fattening farm on day 69 (ND_50_ = 140 and 280, Figure 4 and Figure S1). Neutralizing antibody titers from horizontally infected piglets (*n* = 4) of the same age (69-day-old) were between 35 and 112, while higher neutralizing antibody titers (between 70 and 703) were observed at time of slaughter (day 161, Figure 4). Sera of the pigs from CT affected litters showed various amounts of neutralizing antibodies at the time of slaughter (ND_50_ ≤ 4 to 2798, Figure 4). Nevertheless, levels of neutralizing antibodies were the highest in horizontally infected pigs at the final sampling, with the exception of only two pigs derived from CT affected litters (P-6 and P-13). High neutralizing antibody titers of 2798 and 708 were detected in these single individuals (P-6 and P-13, Figure 4 and Figure S1). In general, neutralizing antibody titers correlated with the presence of E2-specific antibodies (Figure 4a), but there was no apparent correlation with E^rns^-specific antibodies (Figure 4b). Sera of two piglets from CT affected litters (P-2, P-6), which showed high reactivity against the E2 protein and only low reactivity against E^rns^ at day 69, displayed relatively high neutralizing antibody titers of 140 and 280 (Figure 4 and Figure S1). Similarly, sera of a healthy piglet from an affected litter (P-13) and a horizontally infected piglet (P-19), both showing high reactivity only against E2, demonstrated high neutralizing antibody titers of 708 and 352 (Figure 4). Furthermore, sera of two piglets (P-8, P-14) from CT affected litters, which were highly reactive only against E^rns^ but not against E2 at day 69, showed no neutralizing ability (ND_50_ ≤ 4, Figure 4 and Figure S1).

## 4. Discussion

APPV is a putatively globally distributed porcine pathogen associated with congenital tremor (CT) of newborn piglets. APPV is regarded as a potential threat to global swine health. Due to eminent genetic differences, biological properties of APPV can be expected to be different from established pestiviruses. So far, the role of porcine humoral immune response in controlling APPV infection has not been investigated. One major reason for this was the lack of serological tools such as VNT, which is restrained by the difficulties of isolation and propagation of APPV in cell culture [3,4,5]. Replication of APPV in cell lines suitable for isolation of CSFV and other pestiviruses is very limited and inefficient. Consequently, successful virus isolation was reported by only a few research groups [9,13,31]. A cell culture adapted APPV variant, showing improved viral replication in vitro, enabled for the first time an investigation into the relevance of neutralizing antibodies in controlling APPV infections. In addition, establishment of a newly developed E2 based antibody ELISA (this study) together with application of a recently established E^rns^ based antibody ELISA [31] allowed monitoring of the kinetics of antibodies against the putative envelope proteins, which represent the major targets of the humoral immune system in classical pestiviruses.

In the present study, transient infection of gilts during the gestation was observed, which is indicated by low or even negative APPV genome detection at the time point of farrowing. All gilts showed intermediate to high antibody levels against both APPV E^rns^ and E2. Although the test virus used in the VNT (isolate Ger-NRW_L277) was homologous to the outbreak isolate, only moderate titers of neutralizing antibodies (ND_50_ = 28 to 112) were detectable in the majority of sows. Despite close and direct contact of the sows with virus shedding offspring for a period of three weeks, no major titer changes were observed within the first seven weeks after farrowing.

In concordance with the detection of antibodies in the sows, six-day-old piglets showed intermediate to high levels of E^rns^ and E2-specific antibodies. APPV-specific antibody levels declined over time and reached minimal antibody titers at an age of 21 to 48 days (Figure 2 and Figure 3) indicating maternal origin. Presence of maternally derived antibodies (MDA) was monitored in all piglets, independent of their health status. Half-life of porcine MDA is approximately 14 days for immunoglobulin IgG, the major isotype in pig colostrum, and levels gradually decline over time, which is in line with the presented results [33,34,35,36,37]. Previously, presence of MDA was also detected in CT affected piglets until 8 weeks of age [9]. Sera of six-day-old piglets revealed low to moderate neutralizing ability (ND_50_ = 9 to 112, Figure 4), which is in line with neutralizing antibody titers detected in the gilts. Despite the presence of MDA, efficient horizontal infection was observed when 21-day-old APPV genome negative piglets (*n* = 6) were mingled on the flat deck with APPV infected piglets (Figure 1d). Moreover, a single healthy piglet (P-15) from a CT affected litter showed highest antibody titers against both proteins as well as neutralizing antibodies (ND_50_ = 70) and was genome negative at an age of six days. Nevertheless, antibody titers declined very rapidly and this piglet became viremic before entering the flat deck, without apparent clinical symptoms (Figure 1c and Figure S1). In CSFV infections, MDA are considered to provide passive protection to the piglets until development of an active immunity [34,35]. In contrast, MDAs did not confer protection against APPV infection in the present study. This observation can be explained by the limited neutralizing capacity and the rapid decline within the first weeks. Nevertheless, as horizontal APPV infection does not result in any clinical signs, it would usually remain undetected by the farmer.

Horizontally infected piglets (*n* = 6) developed consistently high antibody titers against APPV E2 between days 69 and 161 (Figure 2d). Furthermore, neutralizing antibody titers reached up to 703 at the final sampling (Figure 4), suggesting the establishment of protective immunity against APPV after horizontal infection. Age of piglets at the time of horizontal infection might represent a relevant factor for induction of protective immunity. Since the porcine immune system is fully matured in four-week-old piglets, a stronger antibody response after horizontal APPV infection might be achieved by piglets weaned later than 21 days [33,38]. Recovery from acute CSFV and BVDV infections usually results in long-lasting immunity for several years or even for lifetime [34,35,39]. So far, the duration of protective immunity acquired after APPV infection remains unknown and needs to be investigated in further studies.

The E2 glycoprotein is known to be the immune-dominant antigen of classical pestiviruses such as CSFV and BVDV [27,28]. As E2 is targeted by the majority of neutralizing antibodies, subunit vaccines against CSFV are based on E2 antigen [38]. The E2 glycoprotein was recently suggested to be suitable for vaccine development, assuming that E2 is also the main immunogenic antigen of APPV. A recombinant E2 glycoprotein, which was not described in detail, was used to test the immunogenicity in mice [30]. In the present study, induction of humoral immune response after a field infection was investigated in the natural porcine host for the first time. The observed correlation between the levels of E2-specific antibodies and the neutralizing capacity provide evidence that E2 is the major target for induction of neutralizing antibodies against APPV (Figure 4a). This hypothesis is supported by the neutralizing antibody titers of individual sera being only reactive against the E2 antigen (Figure 4a,b). APPV is genetically highly variable and significantly differs from other pestivirus species. A previous study demonstrated that there is no cross-reactivity of APPV antibody positive samples in established serological assays for diagnosis of pestiviruses [31]. However, the extent of cross-protection of APPV-specific antibodies between different APPV genotypes as well as against other pestiviruses remains unknown and will be investigated in future studies.

In the present study, the great majority of piglets (*n* = 14/15) from CT affected litters were initially APPV genome positive (Figure 1a–c), of which 9 piglets showed clinical signs of CT, suggesting vertical infection of the piglets. APPV genome was detectable in sera from these putatively vertically infected piglets until slaughter and only slowly declined over a period of several months (Figure 1a–c). In contrast, horizontal transmission of APPV at later time points resulted in transient infection and absence of viral genomes at the time point of slaughter (Figure 1d). After the decline of MDA, various levels of E^rns^ and E2-specific antibodies were detected throughout the lifetime in sera of the putatively vertically infected piglets (Figure 2 and Figure 3). All CT affected piglets except one (P-8) showed neutralizing antibody titers at the time of slaughter (Figure 4 and Figure S1). However, a low decrease in APPV genome titer was observed despite the absence of neutralizing antibodies (P-8, Figure 1 and Figure S1). Furthermore, antibodies against E^rns^ became evident, but were no longer detected at the time of slaughter (P-8, Figure 3 and Figure S1).

Pestiviruses are known to induce persistent infection following an intrauterine or early post-natal infection [34,39,40]. Persistently infected (PI) animals are immune tolerant to the persisting viral strain and shed high quantities of virus for life, thus representing critical virus reservoirs [34,39,40]. Detection and elimination of PI animals represents a key element of pestivirus control programs [39,40]. In a previous study, two CT affected piglets were identified to reach sexual maturity while still shedding virus in the absence of NS3-specific antibodies [9]. This is in agreement with the detection of APPV genomes in most piglets from CT affected litters at the time point of slaughter (Figure 1b,c). However, two piglets (P-6, P-13) from CT affected litters developed exceptionally high neutralizing antibody titers and were only transiently infected with APPV; these observations are in contrast to crucial characteristics of persistent infections with other pestiviruses (Figure 4 and Figure S1). In general, the outcome of intrauterine infection with pestiviruses depends largely on the time point of gestation [33]. The observed individual differences in development of antibody response against APPV infection and establishment of persistent versus transient infection might be due to different individual development of the piglets at the time point of infection. The fact that one infected litter can consist of diseased, healthy and also uninfected piglets (P-15) supports the hypothesis of subsequent intrauterine infections that proceed only slowly, a scenario well-known for intrauterine porcine parvovirus infections.

## 5. Conclusions

Subclinical horizontal infection with APPV on the flat deck resulted in the induction of high neutralizing antibody titers, which provide protective immunity mainly based on E2-specific antibodies. Therefore, horizontal and clinically inapparent infections of piglets in the post-weaning period seem to be beneficial to establish solid herd immunity. Introducing APPV naïve sows into the herd (or possibly sows that only had contact with a distinct APPV genotype) might cause re-appearance of CT and is in line with the observation that mainly gilts in their first parity farrow CT affected piglets [5]. Another scenario for the delivery of CT affected piglets is that those gilts may still be viremic when inseminated for the first time. In contrast to persistent infections with classical pestiviruses, APPV genome titers declined in piglets from CT affected litters and a humoral immune response against E^rns^ and E2 was detectable. Further investigations will be needed to understand this phenomenon.

The availability of serological assays including VNT and the results of the present study will help to implement tailored prevention strategies against APPV infections. Effective measures may include testing boars and semen for presence of APPV genomes, determination of the APPV genome and antibody status of gilts, extend acclimatization times of gilts newly introduced in a herd and bringing naïve gilts in contact with APPV to allow induction of humoral immune response against APPV before the insemination.

The present study provides a first insight into the humoral immune response against APPV infections in the natural host. Elucidating the capacity of neutralizing antibody induction after APPV infections will help to understand the epidemiology and pathogenesis of this novel pathogen.

## Supplementary Materials

The following are available online at https://www.mdpi.com/1999-4915/11/10/880/s1, Figure S1: Course of APPV infection and antibody response in individual piglets, Figure S2: Development of a novel APPV Virus Neutralization Test (VNT).
