# Supplementary material for: Characterization of the Humoral Immune Response Induced after Infection with Atypical Porcine Pestivirus (APPV)
Source: Viruses. 2019 Sep 21;11(10):880. doi: 10.3390/v11100880 (PMC6832543; doi:10.3390/v11100880)
Supplement: Supplementary file 1 [file viruses-11-00880-s001.pdf]

Supplementary Materials: Characterization of the Humoral Immune Response Induced After Infection with Atypical Porcine Pestivirus (APPV)

Gökce Nur Cagatay, Denise Meyer, Michael Wendt, Paul Becher and Alexander Postel

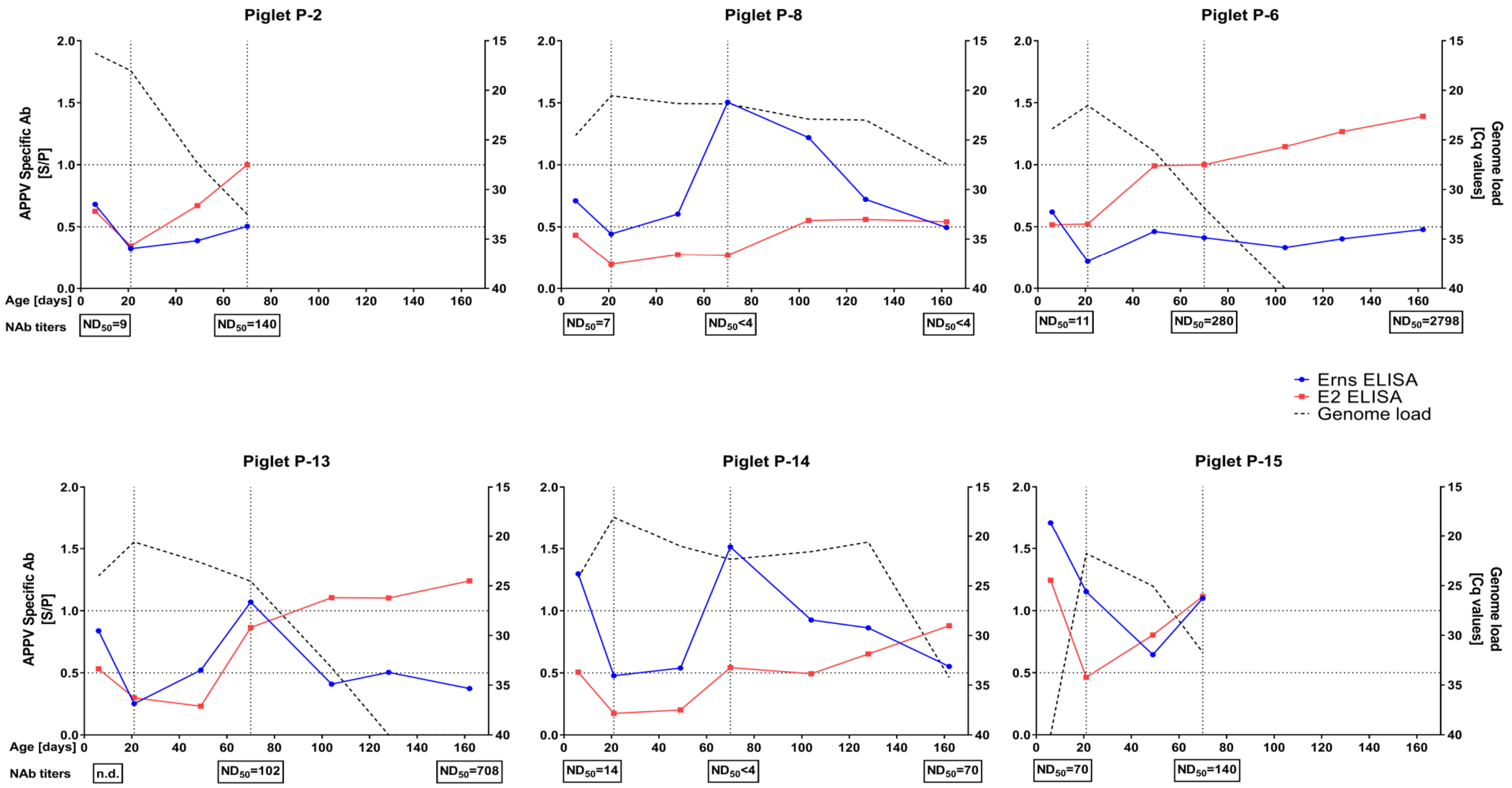

Figure S1. Course of APPV infection and antibody response in individual piglets from CT affected litters. Upper row: CT affected piglets, lower row: healthy piglets from CT affected litters. Vertical dashed lines on day 21 and day 69 indicates the time of weaning and time of transfer to a fattening farm. Horizontal dashed lines indicates the E2 and E^{rns}-specific antibody levels as low, intermediate and high, which are given as S/P values (S/P ≤ 0.5, low; S/P = 0.5- 1.0 intermediate; S/P ≥ 1.0 highly reactive). Neutralizing antibody (nAb) titers are given for days six, 69 and 161. ” n.d.”: not done. Only the piglets, which were discussed individually within the text, are presented. Piglet P-2 is a representative for the CT affected litter 1 that was cross-fostered immediately after birth to a pluriparous sow with a healthy litter. Since various patterns of Ab response are seen in piglets from CT affected litters, two individual examples of diseased (P-6 & P-8) and healthy (P-13 & P-14) piglets of CT affected litters are shown. P-15 is the only piglet from the CT affected litters that was initially genome negative.

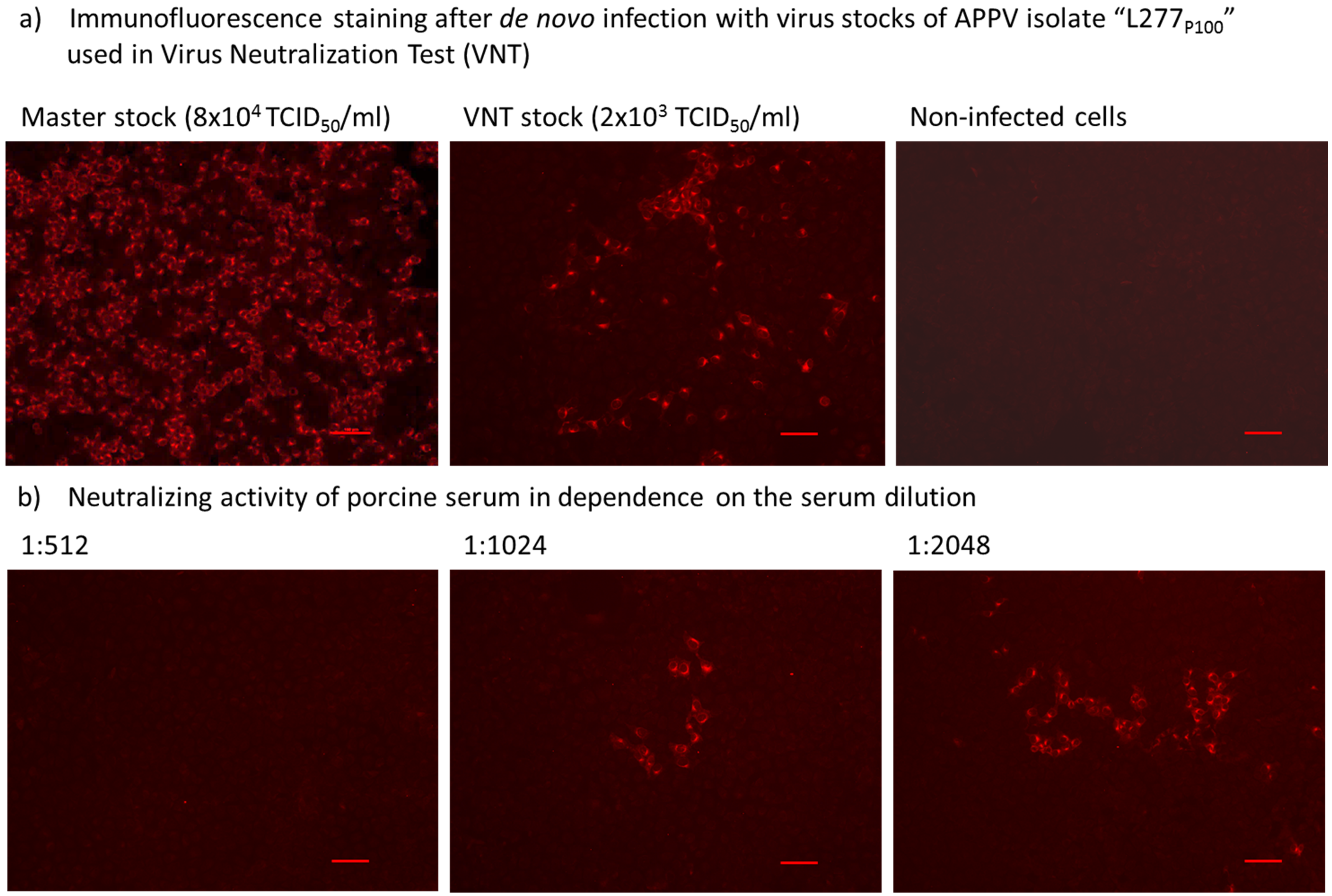

Figure S2. Development of a novel APPV Virus Neutralization Test (VNT). **Panel a)** Immunofluorescence staining after de novo infection with virus stocks of APPV isolate "$L277_{P100}$" used in VNT. SPEV cells were infected simultaneously at the time point of seeding. Cells were heat fixated 3 days post infection for immunofluorescence staining. Non infected cells served as a negative control. **Panel b)** Neutralizing activity of a porcine serum in dependence on the serum dilution. Serial dilutions of a serum sample (used as a control serum in each VNT assay) were performed using Log 2 dilution steps. VNT was performed as described within the manuscript and APPV "$L277_{P100}$" VNT stock (102 $TCID_{50}$/50µl) shown in panel a was used as a test virus. Scale bars indicate 100 µm.
